# The Racer’s Brain – How Domain Expertise is Reflected in the Neural Substrates of Driving

**DOI:** 10.3389/fnhum.2015.00635

**Published:** 2015-11-24

**Authors:** Otto Lappi

**Affiliations:** Cognitive Science & Traffic Research Unit, Institute of Behavioural Sciences, University of HelsinkiHelsinki, Finland

**Keywords:** expertise, driving, race driving, simulators, brain imaging, fMRI

## Abstract

A fundamental question in human brain plasticity is how sensory, motor, and cognitive functions adapt in the process of skill acquisition extended over a period of many years. Recently, there has emerged a growing interest in cognitive neuroscience on studying the functional and structural differences in the brains of elite athletes. Elite performance in sports, music, or the arts, allows us to observe sensorimotor and cognitive performance at the limits of human capability. In this mini-review, we look at driving expertise. The emerging brain imaging literature on the neural substrates of real and simulated driving is reviewed (for the first time), and used as the context for interpreting recent findings on the differences between racing drivers and non-athlete controls. Also the cognitive psychology and cognitive neuroscience of expertise are discussed.

## Introduction

Elite performance in sports, music, or the arts allows us to observe human performance at the limits of the sensorimotor and cognitive capacity of the mind and brain. Recently, interest has emerged in cognitive neuroscience on the functional and structural adaptations in the brains of elite athletes that make possible their exceptional performance (Yarrow et al., 2009). Findings in specific contexts, such as sports, can also teach us more generally about how sensory, motor, and cognitive functions adapt during the process of extended skill acquisition. This may have applications in designing optimal sports coaching methods, but also potentially in overcoming learning disabilities, or in neurological rehabilitation.

**FIGURE 1:**
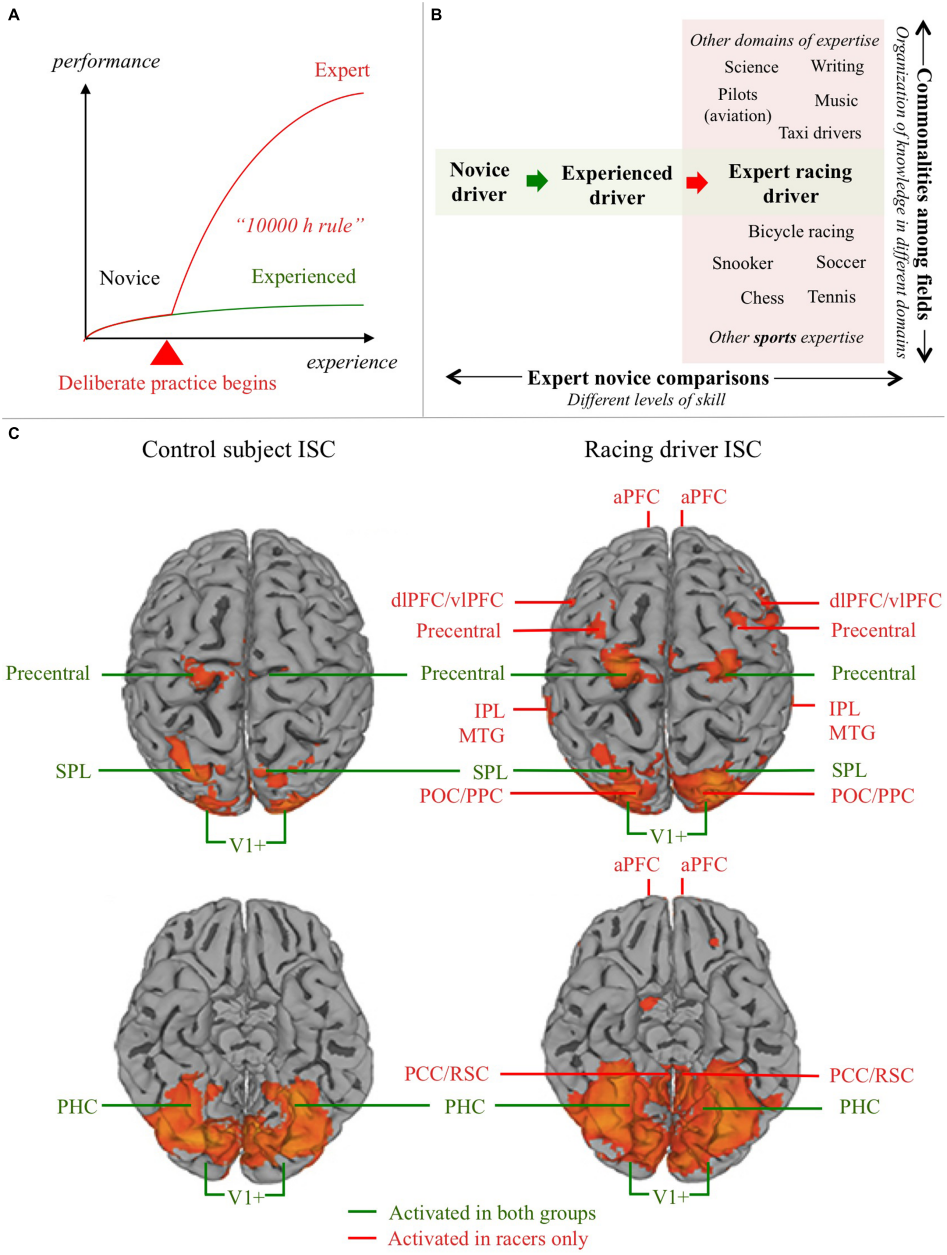
**Domain expertise and the neural substrates of driving**. **(A)** Schematic depiction of the relationship between experience and expertise. Development of expertise differs qualitatively from mere experience: initiating and sustaining deliberate practice is something we are able to do in one or maybe a few domains in our lives. Most skills (such as normal driving) never develop into a form of expertise. 10 000 h of driving experience does not make you an expert, because performance quickly plateaus to a level of merely satisfactory automatic performance. Experts, in contrast, re-invest cognitive capacity (freed by automatization) to improve performance further. **(B)** Understanding the neural substrate of expert performance can be approached in two ways: (i) Commonalities can be looked for in expert brain structure and function in different fields, to seek for general markers of expertise (e.g., different sports, or a sport and fields of expertise more or less similar in terms of cognitive demands); (ii) Experts in a specific domain can be compared to novices, or (as here) experienced non-experts in the domain, in order to understand how domain expertise modifies the neural substrates of task performance. **(C)** Control subject (experienced non-expert) and expert (racing car driver) ISC activation in a semi-active driving task. Based on Bernardi et al., 2014, Figure 1; doi: 10.3389/fnhum.2014.00888, Copyright Bernardi et al. (2014). aPFC, anterior prefrontal cortex; dlPFC, dorsolateral prefrontal cortex; vlPFC, ventrolateral prefrontal cortex; IPL, inferior parietal lobule; SPL, superior parietal lobule; MTG, middle temporal gyrus; POC/PPC, parieto-occipital/posterior parietal cortex; PHC, parahippocampal cortex; PCC/RSC, posterior cingulate/retrosplenial cortex; V1+, occipital visual areas.

Research in the cognitive psychology of expertise has shown that these feats of excellence are not just a reflection of superior innate sensorimotor ability or general intelligence. They are, instead, based on a wealth of domain-specific skills and knowledge, accumulated over years of sustained effort. It is generally agreed that to attain expertise it is necessary to engage in sufficient amount of diligent and well-designed *deliberate practice* (Ericsson et al., 1993). The oft-quoted “10 000 h rule” (**Figure 1A**) states that to attain professional-level expertise in many domains, 10 000 h of engagement in deliberate practice is necessary (but not necessarily sufficient; Hambrick et al., 2014; Lombardo and Deaner, 2014). This translates to engagement in a laborious process of self-improvement for 4 h/day, on average, over a period 10 years.

If expertise is the product of domain-specific knowledge and skills, then it makes sense to ask whether expert performance is made possible by specific, localized neural circuits. And if it is acquired by many years of extensive domain-specific training, one can look for evidence of experience-dependent plasticity in these circuits.

Race driving is a particularly good domain of sports expertise to study, because physically accurate stimuli and motor tasks can be presented in a controlled environment (simulators). Also, the racing driver is tightly harnessed to the vehicle, essentially leaving only eye, neck, shoulder, elbow, wrist, and ankle free to move. And even the highest levels of expertise are exercised through a low-dimensional system of vehicle controls (steering, throttle, brakes).

This mini-review looks at the neural substrates of driving, and the expertise involved in racing. Functional neuroimaging results on the neural substrates driving from the past 15 years are reviewed (the first systematic overview of this literature). This is used as a context to interpret recently observer differences in brain function between racing drivers and non-athlete controls by Bernardi et al. (2014). This approach is predicated on the idea of interpreting exceptional individuals’ brain function in comparison to experienced non-experts. (The other approach would be to seek “loci of excellence” that would be common in all sports or cognitively similar exceptional skills; **Figure 1B**). The thinking is that one should first analyze in detail the sensorimotor and cognitive task requirements of particular domains, and use this to guide the interpretation of the patterns of functional and structural plasticity produced by everyday learning and the rigorous demands of deliberate practice. In time, a more complete picture of what brain processes are specific to each task, and what general principles might be shared between expertise in different domains, will emerge.

## Neural Substrates Of Normal Driving

An overview of the recent brain imaging literature on simulated and real driving in normal subjects is presented in **Table 1.** (Talairach coordinates of peak activation are collected in **Supplementary Table S1**, along with additional details of the experimental tasks and contrasts). The studies were selected on the basis that they should report brain activation in basic steering tasks (i.e., with no complex secondary tasks).

**Table 1:** Brain areas activated by driving tasks.

Reference	[Wal]	[Cal]	[Hor]	[Jeo]	[Sp1]	[Sp2]	[Li]	[Kan]	[Ber]
Method	fMRI	fMRI	PET	PET	fMRI	fMRI	fMRI	fMRI	fMRI
	GLM	ICA			GLM	GLM	MCCA	GLM	ISC
**Left hemisphere**				
Precentral/postcentral	BA4	BA6		BA4	BA4	BA4	BA6	BA4	BA6
	BA1			BA6	BA6	BA6			
	BA3			BA3					
Prefrontal		BA11			BA9	BA44	BA9		BA9
				BA10	BA47			BA45
				BA47				BA47
									BA13
Anterior cingulate		BA24				BA24			BA32
		BA32							
Posterior cingulate/retrosplenial		BA30			BA30		BA29		BA29
					BA31				BA31
Para-hippocampal/hippocampal		BA36		BA35					BA28
		BA37		BA30					BA34
									BA36
									HC
Posterior parietal	BA7	BA7		BA7	BA7	BA7	BA7		BA7
					BA40	BA40	BA40		BA40
uperior temporal								BA41	
Parieto-occipital	BA19	BA17			BA19	BA19	BA18		BA39
		BA18							
		BA19							
		BA7							
Middle temporal						BA37			BA37
						BA39			BA21
									BA22
Occipito-temporal		BA18	BA19			BA19			
		BA19				BA37			
		BA20							
		BA37							
Occipital		BA17		BA17		BA18	BA17	BA19	
		BA18		BA18		BA19	BA18		
		BA19							
Basal ganglia								Put.	CN
Thalamus		N.S.							MDN
Cerebellum	Culm.Ver.	Culm.	Decl.Uvula		Tuber Pyr.	Pyr. Ver-Tuber	Culm.		
**Right hemisphere**				
Precentral/postcentral		BA6	BA6	BA3	BA6	BA4	BA6	BA6	BA6
Precentral/postcentral		BA6	BA6	BA3	BA8	BA6	BA6	BA6	BA6
Precentral/postcentral						BA8	BA6	BA6	BA6
						BA1			
Prefrontal		BA10			BA9	BA9	BA9		BA9
Prefrontal		BA11			BA10	BA10	BA9		BA10
Prefrontal					BA13	BA47	BA9		BA47
Prefrontal					BA47	BA13	BA9		
Anterior cingulate		BA32			BA32				BA24
Posterior cingulate/retrosplenial		BA30		BA24	BA30	BA31	BA29		BA23
				BA31					BA30
Para-hippocampal/hippocampal		BA36							BA20
		BA37							BA36
Posterior parietal	BA7	BA7			BA7	BA7	BA7	BA7	BA40
					BA40		BA40		
					BA40		BA40		
Superior temporal					BA40	BA22			
Parieto-occipital	BA19	BA19	BA17	BA7	BA19		BA18		BA19
		BA7		BA31					BA7
Middle temporal				BA19	BA21	BA37			BA37
				BA39	BA39				
Occipito-temporal		BA18	BA19	BA19	BA18	BA18			
		BA19		BA37	BA18	BA19			
		BA37			BA18	BA21			
Occipital		BA17				BA18	BA17		
		BA18					BA18		
Basal ganglia									CN GP
Thalamus		VLN MDN		VPM		VPL			
Cerebellum	Culm. Ver. Ant. lobe		Ton-sil		Culm. Tuber	Decl. Ver. Culm.	Culm.	Ton-sil	Culm.


*[Wal], Walter et al., 2001; [Cal], Calhoun et al., 2002; [Hor], Horikawa et al., 2005; [Jeo], Jeong et al., 2006; [Sp1], Spiers and Maguire, 2006; [Sp2], Spiers and Maguire, 2007; [Li], Li et al., 2012; [Kan], Kan et al., 2013; [Ber], Bernardi et al., 2014; Put., putamen; CN, Caudate nucleus; MDN, mediodorsal thalamic nucleus; VLN, ventrolateral thalamic nucleus; Pyr., pyramis; Culm., Culmen; Decl., Declive; GLM, General linear model; ICA, Independent component analysis; MCCA, Multiset canonical correlation analysis; ISC, Intersubject correlation. All results are for active simulated driving, except [Jeo] (real driving) and [Ber] (semi–active). For the passive simulated driving results in these studies and (Mader et al., 2009), see **Supplementary Table S1**.*

The most common finding is the activation of premotor frontal areas (BA6/8) and occipital visual areas (BA17/18/19), extending dorsally into medial temporal cortex and occipito-parietal areas (cuneus, precuneus), all the way to the posterior parietal cortex (PPC; BA7 and BA40 in SPL and IPL, respectively). The frontal activation extends to dorsolateral (BA9/46), anterior (BA10), and ventrolateral (BA 44/45) prefrontal cortices, and medial prefrontal and insular areas (BA47/13). Cingulate cortex activation is prevalent in the posterior cingulate/retrosplenial cortex, in the right hemisphere in particular. Within the ventral (occipito-temporal) system, activation is seen in the angular and fusiform gyri. Cerebellar activation at various sites has been observed in most studies, and some studies report activation in the basal ganglia and thalamus, but not all.

The general pattern is reasonably consistent, and the premotor and occipito-parietal activations in particular make sense, as driving is a visuomotor task where steering action needs to be adjusted to observed road geometry. The pattern of results is, however, by no means specific enough to amount to a detailed neurological understanding of the circuitry involved in driving. And the specific pattern of activation seems to depend on task, stimulus, and imaging methods in ways that are not yet well understood.

## Racer vs. Control Comparison

Bernardi et al. (2014) used inter-subject correlation (ISC) to identify group level differences in brain activation between professional racing drivers (*n* = 11) and “naïve” controls (*n* = 11; **Figure 1C**). The advantage of reverse-correlation methods like ISC is that it reveals hemodynamic responses in multiple brain areas based on the synchrony of activation between subjects. It therefore does not require a rigid trial structure, repeating the same stimulus, or *a priori* identification of trigger events. This makes it particularly suitable for designs with naturalistic stimuli that present the subject with continuous streams stimulation, unlike more traditional block designs (Hasson et al., 2004, 2010; Kauppi et al., 2010).

The participants were shown in-car footage of an F1 car driving on official circuits, and were instructed to “imagine themselves driving the racing car”. Brain activity was measured using fMRI, and the BOLD signal time-series were analyzed to find areas that were reliably co-activated within each group (voxelwise Pearson’s correlation, significance testing by permutation test).

Significant activation was observed in both groups, bilaterally, in the visual cortex (V1+, BA 17/18), precentral cortex, posterior parietal cortex (superior parietal lobule, SPL), and parahippocampal cortex (PHC). The racer group showed, in addition, activation in dorsal visual stream (dorsal occipital/PPC), medial temporal gyrus (MTG), lateral prefrontal cortex, and the frontal pole (BA10), and posterior cingulate/retrosplenial cortex (PCC/RSC).

The groupwise activations were compared to reveal brain areas where activity was stimulus-modulated in the racer group significantly more than in the control group. This Racer > Control comparison showed activation in prefrontal cortex (middle frontal gyrus, bilaterally, Broca’s area in the inferior frontal gyrus of the left hemisphere, and right anterior prefrontal cortex), culmen of the right cerebellum, and further bilateral activations in MTG, PPC (supramarginal IPL), anterior cingulate (ACC), PCC/RSC, and caudate nucleus.

## An Integrative View And Theoretical Interpretations

Activation of visual and precentral cortices was present in both groups, consistent with the previous literature. Occipital/occipito-temporal activation makes sense, as the task is to watch a video. Precentral activation, as well as the activation in the PPC (SPL), in both groups may indicate motor preparation, motor imagery, and visuo-spatial attention, reflecting the subjects’ engagement (this was a semi-active task, see below).

Fronto-parietal (dlPFC, IPL) responses were observed only in the Racer group. However, dlPFC/IPL activation has been observed also in normal subjects, by Walter et al. (2001), Calhoun et al. (2002) and Li et al. (2012). The roles of these areas are not obvious – this activation may be related to motor planning, eye movement behavior (FEF, PEF), and/or attentional strategies, which could all be more systematic – and therefore synchronized at group level – in the racers. Spiers and Maguire (2006, 2007) observed IPL and dlPFC activation during action planning and route planning.

Dorsal visual stream (precuneus) and parietal activation was more widespread in the racers. Could this be taken as an indication of higher level of engagement and/or richer visual representations in the expert group? With this type of reasoning one must proceed with caution: one might expect from common sense that experts would bring more “brainpower” to bear on the task. But in fact, what one often finds is that increased proficiency in a task leads to reduced, “more focused”, brain activation. This phenomenon has been dubbed neural efficiency (Haier et al., 1992), a perhaps counterintuitive *inverse* relationship between cognitive performance and brain metabolic activity. The basis for this is not yet understood at the level of mechanistic explanation (Poldrack, 2015), but could be due to more selective activation (only the relevant circuits), or reduced “processing cost” (less activation for a given task in the circuits involved). For expert–control comparisons, this would seem to suggest that for the same task (within the domain of expertise), an expert’s brain should show less activation (and indeed, see Bernardi et al., 2013).

So, why the more widespread pattern of activation in the racer group? One reason may be the use of ISC, which measures inter-subject synchrony – not individual signal change/metabolic level. Another reason may be that while the *stimulus* and *instruction* were the same for the two groups, the *performed tasks* may still be different, in terms of cognitive processes engaged. In direct comparisons, it is necessary to use relatively simple tasks (so that even the non-expert can perform them qualitatively similarly to the expert). By contrast, with a fairly naturalistic task (such as semi-active engagement used here), the task and instruction underdetermine the strategy used. While neural efficiency involves reduced activation in expert performance *in a given task*, in complex naturalistic tasks the knowledge and skill of the subjects *partly determines the task itself*, in terms of cognitive processes engaged. Thus, neural efficiency is best assessed by restricting the task to relatively simple tasks – as opposed to investigating tasks that are more typical for the experts.

Now, “naïve” participants may have a large amount of driving experience (i.e., exposure to road environments similar enough to the racetrack to engage with the task). However, they are unlikely to have developed the kind of rich and extensive domain knowledge that underlies expert cognitive and sensorimotor performance. This in turn may make the “viewing task” qualitatively different for the expert group. Expertise in racing is a result of intense training at a high level of cognitive and physical demand, which can result in qualitatively different skill sets to be employed in the (racing car) driving task. Therefore, it is plausible that when the race drivers view the in-car footage, they are not merely engaged in mentally simulating *steering* in the direction of the bends – i.e., lower-level sensorimotor control routines sufficient for everyday driving – but bring to the task a number of not yet fully understood cognitive skills, required to “read” the visual information from road geometry and landmarks at racing speeds. And hence recruit more brain areas. The situation is analogous to the expert taxi–driver studies (Spiers and Maguire, 2007, 2008), where the simulated road environment is used to evoke the experts’ rich high-level knowledge schemata.

In this light, perhaps the most interesting results pertain to the pattern in the PCC/RSC (BA30) and PHC (BA36). Activation in the PHC – bilateral in both groups – is to be expected with stimuli depicting 3D scenes based on prior work on (non-driving) scene perception imaging studies (Aguirre et al., 1996; Epstein and Kanwisher, 1998). But whereas the PHC is thought to encode viewpoint-dependent scene information, the retrosplenial cortex has been implicated in viewpoint-independent integration of scene information – “piecing together” the scene from locally observed snapshots (Spiers and Maguire, 2006, 2007, observed RSC activation in relation to spontaneous navigation and action planning; see also Iaria et al., 2007; Park and Chun, 2009; Vann et al., 2009). That these areas show up in the Racer > Control contrast may give us a window into the specific ability to “read” a road scene for 3D information relevant to motor planning – an essential skill in race driving. Notably, RSC structural differences were predictive of real-world performance in the Racer group.

Posterior parietal cortex and PFC are also activated in the Racers group. The RSC is closely anatomically and functionally connected to posterior parietal areas (involved in egocentric frame of reference transformations; Andersen et al., 1993; Pouget et al., 2002; McGuire and Sabes, 2009; Crawford et al., 2011), to the hippocampal complex (involved in construction of allocentric cognitive maps; O’Keefe and Nadel, 1978), and the prefrontal cortex (involved in planning and monitoring of complex actions, Ramnani and Owen, 2004). An intriguing possibility – though at this point speculative – is that this pattern reflects higher-level of cognitive task-organization in the experts. The vlPFC (Broca’s area) and dlPFC/aPFC have been implicated complex hierarchical organization in language and music (Patel, 2003), and the organization of hierarchical goals (Fuster, 2001; Koechlin et al., 2003; Petrides, 2005; Koechlin and Hyafil, 2007; Badre, 2008; Botvinick, 2008). Could the prefrontal–parietal–retrosplenial–hippocampal network be involved in processing a hierarchical representation of the driving line in terms of (loco)motor subgoals? This would mean that while the virtual driving task would be essentially path following for the controls, for the experts the planning of driving line would closely resemble a *chunking* process found to underlie pattern recognition, memory, and decision-making in many domains of skill and expertise (Cooper and Shallice, 2000; Gobet et al., 2001).

## Open Issues And Future Directions

One essential factor that is not yet well understood is how the task and the instruction given may affect brain responses. Almost all the studies reviewed are “simulated driving” tasks (except the Jeong et al., 2006 PET study). The subject observes or controls a virtual car in a game environment. Such tasks may be *active* (controlling steering and/or speed of simulated ego-vehicle) or *semi-active* (actively imagining one is driving the ego-vehicle, but no overt movements). These contrast with “passive simulated driving” (viewing an in-car movie from real or simulated driving, as if one were the passenger rather than the driver). Which brain areas are activated by “simulated driving” can then depend on the contrast: in (semi)active tasks the active condition can be contrasted with passive viewing of the moving road scene (or a control stimulus), whereas in passive tasks the passive condition can be “driving”, compared to a non-driving control task. Thus, “driving” can be sometimes active > control, sometimes passive > control, but sometimes it can be active > passive.

Another factor is eye movement. Many of the areas activated in driving are also areas implicated in the control of saccadic and pursuit eye movements (Munoz, 2002; Krauzlis, 2004; Pierrot-Deseilligny et al., 2004), and coordinated and timely information pick-up is essential in many domains of skill. Differences in eye movement patterns caused by differences in the task, display, or subject skill may underpin some of the fronto-parietal activity differences (frontal and parietal eye-fields, FEF, and PEF, respectively). Differences in the way the gaze samples visual information create different retinal inputs, which can in turn produce differences in cortical processing downstream.

In expert vs. experienced non-expert comparisons, it would also be desirable to control the relevant background experience of the controls in more detail. Only sports experience was controlled by Bernardi et al. (2014), not prior exposure to the stimuli (in-car footage is commonly broadcast in televised Formula One events, and virtual models of the tracks can be driven in commercial games). The familiarity of the circuits to the racers is thus confounded with racing-specific skill. Recognition of familiar landmarks will elicit navigational memory in experts, but if some “naïve” controls are familiar with some circuits (through watching TV or playing videogames) this could mask group differences.

Racing game experience is probably especially relevant. If some “naïve” controls have played on virtual versions of the tracks, they may have developed *some* of the cognitive strategies of the professional racers. Comparison between truly naïve controls (but experienced drivers), experienced computer gamers, and real racers would make for an interesting research question in itself. This would control for familiarity and cognitive skills that can be learned in simulators, and might pinpoint specific adaptations in real racing, and begin to tease apart the component skills in “driving” and “racing”.

Of course, non-specific demographic control variables are important, too. Biological and socio-economical heterogeneity in the control group, and matching for ethnicity, socio-economic background, and body mass index are important to consider. But more to the point, by judicious use of controls, it may in fact be possible to better isolate the component skills. This means analysing “expertise” in terms of component capacities, not treating it as a monolithic state variable (and also clearly differentiating it from mere *experience* without deliberate practice).

## Conclusion

We have discussed the expert racer brain activation in relation to known neural substrates of normal driving, and in the contexts of the cognitive psychology of expertise, and the representation of navigational space and complex motor action. The rationale is that the brain substrates of expert driving should first be understood in relation to normal driving (and tasks with similar cognitive components, even if they are not related to driving/sports). Comparison of the similarities and differences in the underpinnings of excellence in different sports can then proceed on a more secure footing.

The more widespread pattern of activation in racers (when semi-actively watching competitive driving from an egocentric perspective) could be due to more homogeneous synchronization of cognitive (and/or oculomotor) processes in the racer group. It may also be that with the development of cognitive expertise in racing, “the driving task” (i.e., the set of skills and cognitive operations involved) may have qualitatively changed. Whereas for a naïve participant steering a series of bends may effectively be reduced to a simple path-following visuomotor routine (even more so observing a video of a car steering a series of bends), to the expert with detailed survey knowledge of the track and a deep understanding of cornering techniques (cued by landmarks), many additional cognitive operations may be performed. This way, the pattern of activation itself can perhaps give us clues about the organization of the cognitive operations involved in racing.

## Conflict of Interest Statement

The author declares that the research was conducted in the absence of any commercial or financial relationships that could be construed as a potential conflict of interest.

## Supplementary Material

TABLE S1 **Talairach coordinates from the studies in **Table 1**, as well as information on the contrasts and additional results for passive conditions**.
